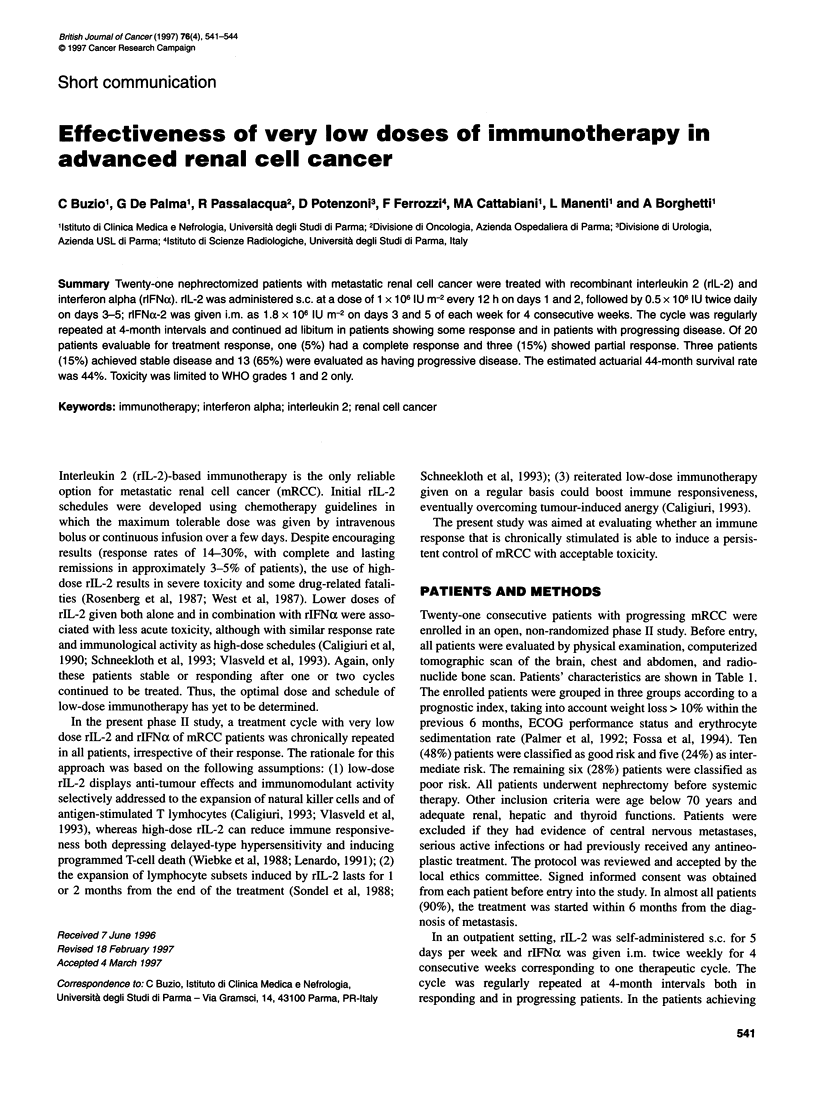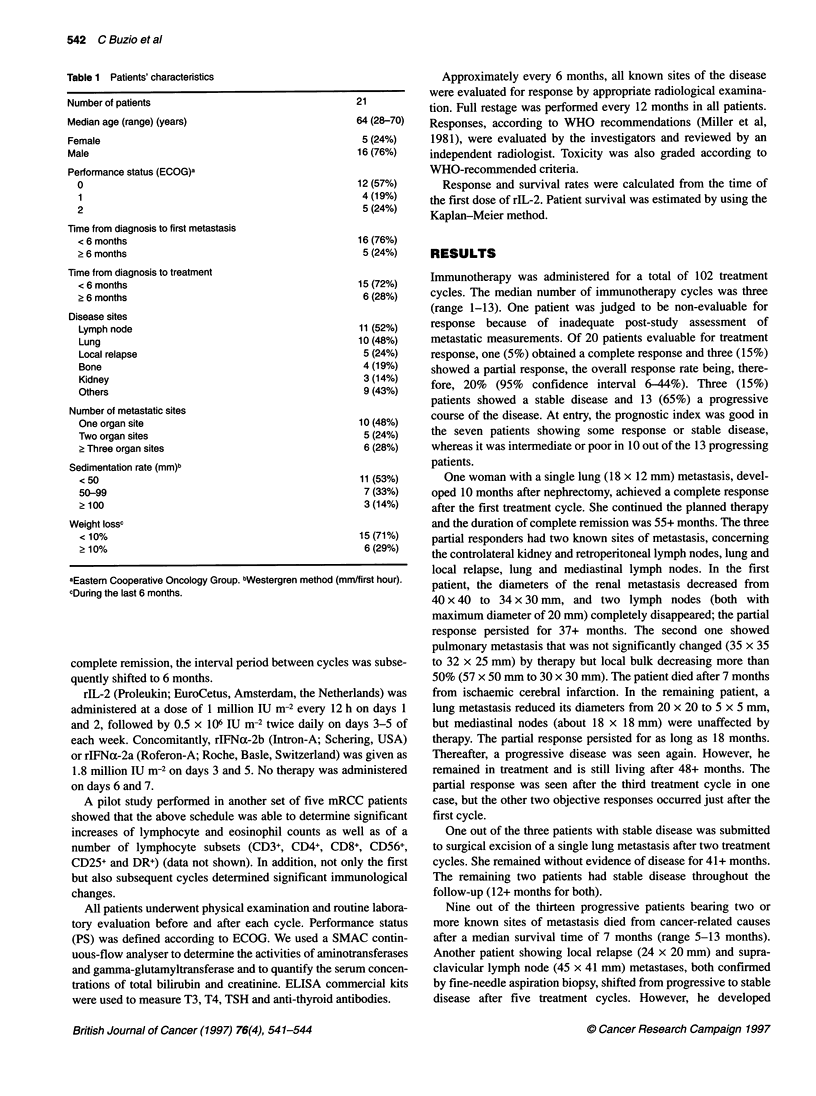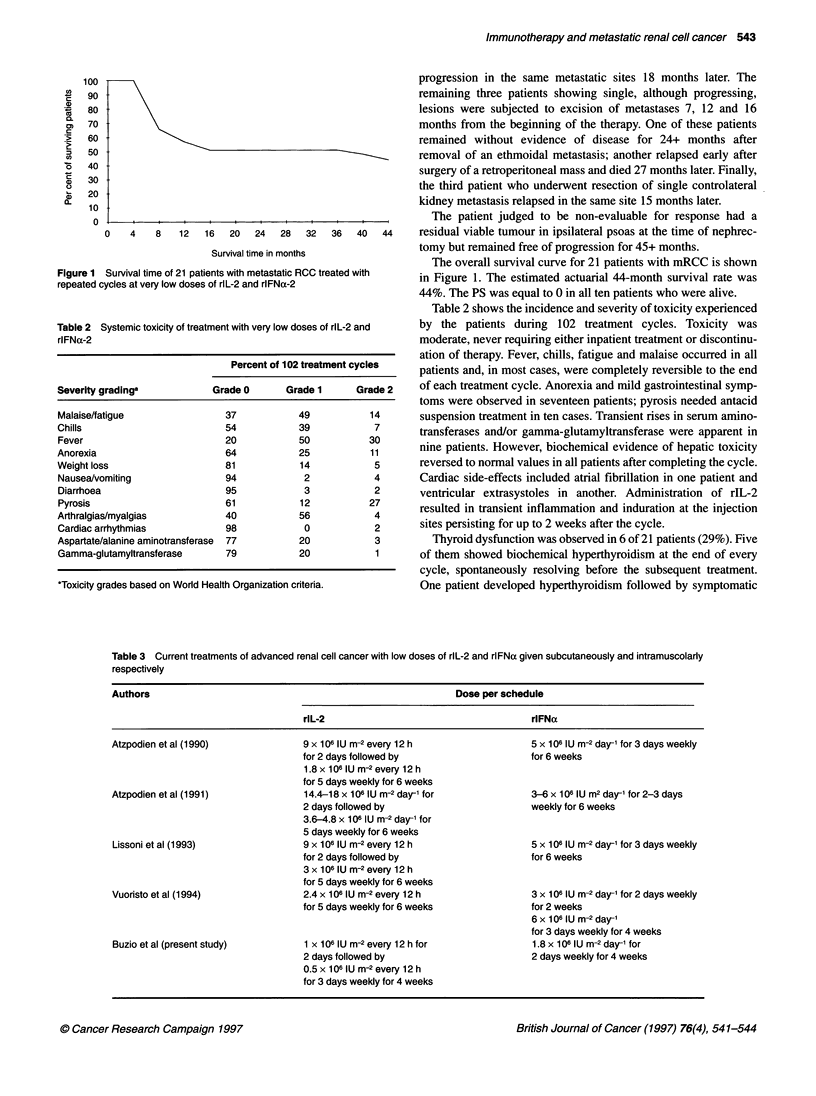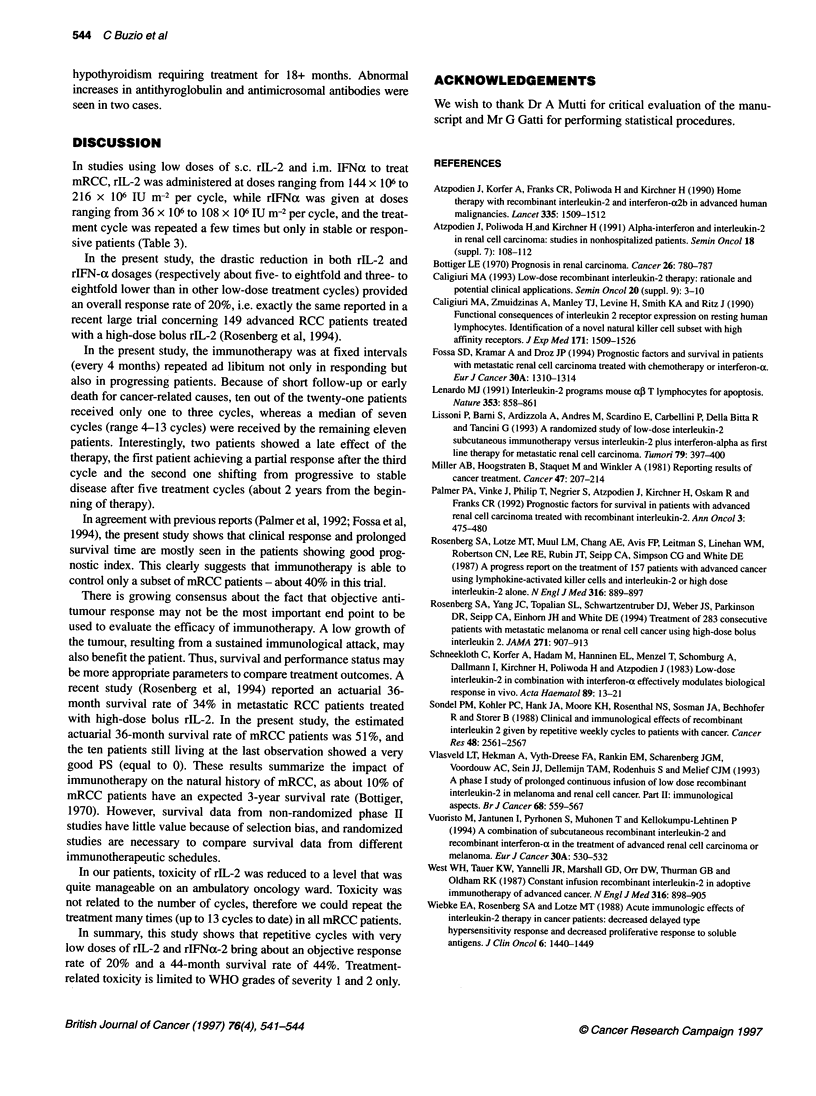# Effectiveness of very low doses of immunotherapy in advanced renal cell cancer.

**DOI:** 10.1038/bjc.1997.422

**Published:** 1997

**Authors:** C. Buzio, G. De Palma, R. Passalacqua, D. Potenzoni, F. Ferrozzi, M. A. Cattabiani, L. Manenti, A. Borghetti

**Affiliations:** Istituto di Clinica Medica e Nefrologia, UniversitÃ degli Studi di Parma, Italy.

## Abstract

Twenty-one nephrectomized patients with metastatic renal cell cancer were treated with recombinant interleukin 2 (rlL-2) and interferon alpha (rIFN alpha). rIL-2 was administered s.c. at a dose of 1 x 10(6) IU m(-2) every 12 h on days 1 and 2, followed by 0.5 x 10(6) IU twice daily on days 3-5; rIFN alpha-2 was given i.m. as 1.8 x 10(6) IU m(-2) on days 3 and 5 of each week for 4 consecutive weeks. The cycle was regularly repeated at 4-month intervals and continued ad libitum in patients showing some response and in patients with progressing disease. Of 20 patients evaluable for treatment response, one (5%) had a complete response and three (15%) showed partial response. Three patients (15%) achieved stable disease and 13 (65%) were evaluated as having progressive disease. The estimated actuarial 44-month survival rate was 44%. Toxicity was limited to WHO grades 1 and 2 only.


British Journal of Cancer (1997) 76(4), 541–544
© 1997 Cancer Research Campaign

Short communication

### Effectiveness of very low doses of immunotherapy in advanced renal cell cancer

C Buzio[1], G De Palma[1], R Passalacqua[2], D Potenzoni[3], F Ferrozzi[4], MA Cattabiani[1], L Manenti[1] and A Borghetti[1]

[1]Istituto di Clinica Medica e Nefrologia, Università degli Studi di Parma; [2]Divisione di Oncologia, Azienda Ospedaliera di Parma; [3]Divisione di Urologia, Azienda USL di Parma; [4]Istituto di Scienze Radiologiche, Università degli Studi di Parma, Italy

**Summary** Twenty-one nephrectomized patients with metastatic renal cell cancer were treated with recombinant interleukin 2 (rIL-2) and interferon alpha (rIFNα). rIL-2 was administered s.c. at a dose of $1 \times 10^6$ IU $m^{-2}$ every 12 h on days 1 and 2, followed by $0.5 \times 10^6$ IU twice daily on days 3–5; rIFNα-2 was given i.m. as $1.8 \times 10^6$ IU $m^{-2}$ on days 3 and 5 of each week for 4 consecutive weeks. The cycle was regularly repeated at 4-month intervals and continued ad libitum in patients showing some response and in patients with progressing disease. Of 20 patients evaluable for treatment response, one (5%) had a complete response and three (15%) showed partial response. Three patients (15%) achieved stable disease and 13 (65%) were evaluated as having progressive disease. The estimated actuarial 44-month survival rate was 44%. Toxicity was limited to WHO grades 1 and 2 only.

**Keywords:** immunotherapy; interferon alpha; interleukin 2; renal cell cancer

Interleukin 2 (rIL-2)-based immunotherapy is the only reliable option for metastatic renal cell cancer (mRCC). Initial rIL-2 schedules were developed using chemotherapy guidelines in which the maximum tolerable dose was given by intravenous bolus or continuous infusion over a few days. Despite encouraging results (response rates of 14–30%, with complete and lasting remissions in approximately 3–5% of patients), the use of high-dose rIL-2 results in severe toxicity and some drug-related fatalities (Rosenberg et al, 1987; West et al, 1987). Lower doses of rIL-2 given both alone and in combination with rIFNα were associated with less acute toxicity, although with similar response rate and immunological activity as high-dose schedules (Caligiuri et al, 1990; Schneekloth et al, 1993; Vlasveld et al, 1993). Again, only these patients stable or responding after one or two cycles continued to be treated. Thus, the optimal dose and schedule of low-dose immunotherapy has yet to be determined.

In the present phase II study, a treatment cycle with very low dose rIL-2 and rIFNα of mRCC patients was chronically repeated in all patients, irrespective of their response. The rationale for this approach was based on the following assumptions: (1) low-dose rIL-2 displays anti-tumour effects and immunomodulant activity selectively addressed to the expansion of natural killer cells and of antigen-stimulated T lymhocytes (Caligiuri, 1993; Vlasveld et al, 1993), whereas high-dose rIL-2 can reduce immune responsiveness both depressing delayed-type hypersensitivity and inducing programmed T-cell death (Wiebke et al, 1988; Lenardo, 1991); (2) the expansion of lymphocyte subsets induced by rIL-2 lasts for 1 or 2 months from the end of the treatment (Sondel et al, 1988; Schneekloth et al, 1993); (3) reiterated low-dose immunotherapy given on a regular basis could boost immune responsiveness, eventually overcoming tumour-induced anergy (Caligiuri, 1993).

The present study was aimed at evaluating whether an immune response that is chronically stimulated is able to induce a persistent control of mRCC with acceptable toxicity.

*Received 7 June 1996*
*Revised 18 February 1997*
*Accepted 4 March 1997*

*Correspondence to:* C Buzio, Istituto di Clinica Medica e Nefrologia, Università degli Studi di Parma – Via Gramsci, 14, 43100 Parma, PR-Italy

### PATIENTS AND METHODS

Twenty-one consecutive patients with progressing mRCC were enrolled in an open, non-randomized phase II study. Before entry, all patients were evaluated by physical examination, computerized tomographic scan of the brain, chest and abdomen, and radionuclide bone scan. Patients' characteristics are shown in Table 1. The enrolled patients were grouped in three groups according to a prognostic index, taking into account weight loss > 10% within the previous 6 months, ECOG performance status and erythrocyte sedimentation rate (Palmer et al, 1992; Fossa et al, 1994). Ten (48%) patients were classified as good risk and five (24%) as intermediate risk. The remaining six (28%) patients were classified as poor risk. All patients underwent nephrectomy before systemic therapy. Other inclusion criteria were age below 70 years and adequate renal, hepatic and thyroid functions. Patients were excluded if they had evidence of central nervous metastases, serious active infections or had previously received any antineoplastic treatment. The protocol was reviewed and accepted by the local ethics committee. Signed informed consent was obtained from each patient before entry into the study. In almost all patients (90%), the treatment was started within 6 months from the diagnosis of metastasis.

In an outpatient setting, rIL-2 was self-administered s.c. for 5 days per week and rIFNα was given i.m. twice weekly for 4 consecutive weeks corresponding to one therapeutic cycle. The cycle was regularly repeated at 4-month intervals both in responding and in progressing patients. In the patients achieving

Table 1 Patients' characteristics

| | |
|---|---|
| Number of patients | 21 |
| Median age (range) (years) | 64 (28–70) |
| Female | 5 (24%) |
| Male | 16 (76%) |
| Performance status (ECOG)[a] | |
| 0 | 12 (57%) |
| 1 | 4 (19%) |
| 2 | 5 (24%) |
| Time from diagnosis to first metastasis | |
| < 6 months | 16 (76%) |
| ≥ 6 months | 5 (24%) |
| Time from diagnosis to treatment | |
| < 6 months | 15 (72%) |
| ≥ 6 months | 6 (28%) |
| Disease sites | |
| Lymph node | 11 (52%) |
| Lung | 10 (48%) |
| Local relapse | 5 (24%) |
| Bone | 4 (19%) |
| Kidney | 3 (14%) |
| Others | 9 (43%) |
| Number of metastatic sites | |
| One organ site | 10 (48%) |
| Two organ sites | 5 (24%) |
| ≥ Three organ sites | 6 (28%) |
| Sedimentation rate (mm)[b] | |
| < 50 | 11 (53%) |
| 50–99 | 7 (33%) |
| ≥ 100 | 3 (14%) |
| Weight loss[c] | |
| < 10% | 15 (71%) |
| ≥ 10% | 6 (29%) |

[a]Eastern Cooperative Oncology Group. [b]Westergren method (mm/first hour). [c]During the last 6 months.

complete remission, the interval period between cycles was subsequently shifted to 6 months.

rIL-2 (Proleukin; EuroCetus, Amsterdam, the Netherlands) was administered at a dose of 1 million IU $m^{-2}$ every 12 h on days 1 and 2, followed by $0.5 \times 10^6$ IU $m^{-2}$ twice daily on days 3–5 of each week. Concomitantly, rIFNα-2b (Intron-A; Schering, USA) or rIFNα-2a (Roferon-A; Roche, Basle, Switzerland) was given as 1.8 million IU $m^{-2}$ on days 3 and 5. No therapy was administered on days 6 and 7.

A pilot study performed in another set of five mRCC patients showed that the above schedule was able to determine significant increases of lymphocyte and eosinophil counts as well as of a number of lymphocyte subsets ($CD3^+$, $CD4^+$, $CD8^+$, $CD56^+$, $CD25^+$ and $DR^+$) (data not shown). In addition, not only the first but also subsequent cycles determined significant immunological changes.

All patients underwent physical examination and routine laboratory evaluation before and after each cycle. Performance status (PS) was defined according to ECOG. We used a SMAC continuous-flow analyser to determine the activities of aminotransferases and gamma-glutamyltransferase and to quantify the serum concentrations of total bilirubin and creatinine. ELISA commercial kits were used to measure T3, T4, TSH and anti-thyroid antibodies.

Approximately every 6 months, all known sites of the disease were evaluated for response by appropriate radiological examination. Full restage was performed every 12 months in all patients. Responses, according to WHO recommendations (Miller et al, 1981), were evaluated by the investigators and reviewed by an independent radiologist. Toxicity was also graded according to WHO-recommended criteria.

Response and survival rates were calculated from the time of the first dose of rIL-2. Patient survival was estimated by using the Kaplan–Meier method.

### RESULTS

Immunotherapy was administered for a total of 102 treatment cycles. The median number of immunotherapy cycles was three (range 1–13). One patient was judged to be non-evaluable for response because of inadequate post-study assessment of metastatic measurements. Of 20 patients evaluable for treatment response, one (5%) obtained a complete response and three (15%) showed a partial response, the overall response rate being, therefore, 20% (95% confidence interval 6–44%). Three (15%) patients showed a stable disease and 13 (65%) a progressive course of the disease. At entry, the prognostic index was good in the seven patients showing some response or stable disease, whereas it was intermediate or poor in 10 out of the 13 progressing patients.

One woman with a single lung (18 × 12 mm) metastasis, developed 10 months after nephrectomy, achieved a complete response after the first treatment cycle. She continued the planned therapy and the duration of complete remission was 55+ months. The three partial responders had two known sites of metastasis, concerning the controlateral kidney and retroperitoneal lymph nodes, lung and local relapse, lung and mediastinal lymph nodes. In the first patient, the diameters of the renal metastasis decreased from 40 × 40 to 34 × 30 mm, and two lymph nodes (both with maximum diameter of 20 mm) completely disappeared; the partial response persisted for 37+ months. The second one showed pulmonary metastasis that was not significantly changed (35 × 35 to 32 × 25 mm) by therapy but local bulk decreasing more than 50% (57 × 50 mm to 30 × 30 mm). The patient died after 7 months from ischaemic cerebral infarction. In the remaining patient, a lung metastasis reduced its diameters from 20 × 20 to 5 × 5 mm, but mediastinal nodes (about 18 × 18 mm) were unaffected by therapy. The partial response persisted for as long as 18 months. Thereafter, a progressive disease was seen again. However, he remained in treatment and is still living after 48+ months. The partial response was seen after the third treatment cycle in one case, but the other two objective responses occurred just after the first cycle.

One out of the three patients with stable disease was submitted to surgical excision of a single lung metastasis after two treatment cycles. She remained without evidence of disease for 41+ months. The remaining two patients had stable disease throughout the follow-up (12+ months for both).

Nine out of the thirteen progressive patients bearing two or more known sites of metastasis died from cancer-related causes after a median survival time of 7 months (range 5–13 months). Another patient showing local relapse (24 × 20 mm) and supraclavicular lymph node (45 × 41 mm) metastases, both confirmed by fine-needle aspiration biopsy, shifted from progressive to stable disease after five treatment cycles. However, he developed

© Cancer Research Campaign 1997

**Figure 1** Survival time of 21 patients with metastatic RCC treated with repeated cycles at very low doses of rIL-2 and rIFNα-2

**Table 2** Systemic toxicity of treatment with very low doses of rIL-2 and rIFNα-2

| | Percent of 102 treatment cycles | | |
|---|---|---|---|
| **Severity grading**[a] | **Grade 0** | **Grade 1** | **Grade 2** |
| Malaise/fatigue | 37 | 49 | 14 |
| Chills | 54 | 39 | 7 |
| Fever | 20 | 50 | 30 |
| Anorexia | 64 | 25 | 11 |
| Weight loss | 81 | 14 | 5 |
| Nausea/vomiting | 94 | 2 | 4 |
| Diarrhoea | 95 | 3 | 2 |
| Pyrosis | 61 | 12 | 27 |
| Arthralgias/myalgias | 40 | 56 | 4 |
| Cardiac arrhythmias | 98 | 0 | 2 |
| Aspartate/alanine aminotransferase | 77 | 20 | 3 |
| Gamma-glutamyltransferase | 79 | 20 | 1 |

[a]Toxicity grades based on World Health Organization criteria.

progression in the same metastatic sites 18 months later. The remaining three patients showing single, although progressing, lesions were subjected to excision of metastases 7, 12 and 16 months from the beginning of the therapy. One of these patients remained without evidence of disease for 24+ months after removal of an ethmoidal metastasis; another relapsed early after surgery of a retroperitoneal mass and died 27 months later. Finally, the third patient who underwent resection of single controlateral kidney metastasis relapsed in the same site 15 months later.

The patient judged to be non-evaluable for response had a residual viable tumour in ipsilateral psoas at the time of nephrectomy but remained free of progression for 45+ months.

The overall survival curve for 21 patients with mRCC is shown in Figure 1. The estimated actuarial 44-month survival rate was 44%. The PS was equal to 0 in all ten patients who were alive.

Table 2 shows the incidence and severity of toxicity experienced by the patients during 102 treatment cycles. Toxicity was moderate, never requiring either inpatient treatment or discontinuation of therapy. Fever, chills, fatigue and malaise occurred in all patients and, in most cases, were completely reversible to the end of each treatment cycle. Anorexia and mild gastrointestinal symptoms were observed in seventeen patients; pyrosis needed antacid suspension treatment in ten cases. Transient rises in serum aminotransferases and/or gamma-glutamyltransferase were apparent in nine patients. However, biochemical evidence of hepatic toxicity reversed to normal values in all patients after completing the cycle. Cardiac side-effects included atrial fibrillation in one patient and ventricular extrasystoles in another. Administration of rIL-2 resulted in transient inflammation and induration at the injection sites persisting for up to 2 weeks after the cycle.

Thyroid dysfunction was observed in 6 of 21 patients (29%). Five of them showed biochemical hyperthyroidism at the end of every cycle, spontaneously resolving before the subsequent treatment. One patient developed hyperthyroidism followed by symptomatic

**Table 3** Current treatments of advanced renal cell cancer with low doses of rIL-2 and rIFNα given subcutaneously and intramuscolarly respectively

| **Authors** | **Dose per schedule** | |
|---|---|---|
| | **rIL-2** | **rIFNα** |
| Atzpodien et al (1990) | $9 \times 10^6$ IU m$^{-2}$ every 12 h for 2 days followed by $1.8 \times 10^6$ IU m$^{-2}$ every 12 h for 5 days weekly for 6 weeks | $5 \times 10^6$ IU m$^{-2}$ day$^{-1}$ for 3 days weekly for 6 weeks |
| Atzpodien et al (1991) | 14.4–18 $\times 10^6$ IU m$^{-2}$ day$^{-1}$ for 2 days followed by 3.6–4.8 $\times 10^6$ IU m$^{-2}$ day$^{-1}$ for 5 days weekly for 6 weeks | 3–6 $\times 10^6$ IU m$^2$ day$^{-1}$ for 2–3 days weekly for 6 weeks |
| Lissoni et al (1993) | $9 \times 10^6$ IU m$^{-2}$ every 12 h for 2 days followed by $3 \times 10^6$ IU m$^{-2}$ every 12 h for 5 days weekly for 6 weeks | $5 \times 10^6$ IU m$^{-2}$ day$^{-1}$ for 3 days weekly for 6 weeks |
| Vuoristo et al (1994) | $2.4 \times 10^6$ IU m$^{-2}$ every 12 h for 5 days weekly for 6 weeks | $3 \times 10^6$ IU m$^{-2}$ day$^{-1}$ for 2 days weekly for 2 weeks $6 \times 10^6$ IU m$^{-2}$ day$^{-1}$ for 3 days weekly for 4 weeks |
| Buzio et al (present study) | $1 \times 10^6$ IU m$^{-2}$ every 12 h for 2 days followed by $0.5 \times 10^6$ IU m$^{-2}$ every 12 h for 3 days weekly for 4 weeks | $1.8 \times 10^6$ IU m$^{-2}$ day$^{-1}$ for 2 days weekly for 4 weeks |

*© Cancer Research Campaign 1997*

hypothyroidism requiring treatment for 18+ months. Abnormal increases in antithyroglobulin and antimicrosomal antibodies were seen in two cases.

### DISCUSSION

In studies using low doses of s.c. rIL-2 and i.m. IFNα to treat mRCC, rIL-2 was administered at doses ranging from $144 \times 10^6$ to $216 \times 10^6$ IU $m^{-2}$ per cycle, while rIFNα was given at doses ranging from $36 \times 10^6$ to $108 \times 10^6$ IU $m^{-2}$ per cycle, and the treatment cycle was repeated a few times but only in stable or responsive patients (Table 3).

In the present study, the drastic reduction in both rIL-2 and rIFN-α dosages (respectively about five- to eightfold and three- to eightfold lower than in other low-dose treatment cycles) provided an overall response rate of 20%, i.e. exactly the same reported in a recent large trial concerning 149 advanced RCC patients treated with a high-dose bolus rIL-2 (Rosenberg et al, 1994).

In the present study, the immunotherapy was at fixed intervals (every 4 months) repeated ad libitum not only in responding but also in progressing patients. Because of short follow-up or early death for cancer-related causes, ten out of the twenty-one patients received only one to three cycles, whereas a median of seven cycles (range 4–13 cycles) were received by the remaining eleven patients. Interestingly, two patients showed a late effect of the therapy, the first patient achieving a partial response after the third cycle and the second one shifting from progressive to stable disease after five treatment cycles (about 2 years from the beginning of therapy).

In agreement with previous reports (Palmer et al, 1992; Fossa et al, 1994), the present study shows that clinical response and prolonged survival time are mostly seen in the patients showing good prognostic index. This clearly suggests that immunotherapy is able to control only a subset of mRCC patients – about 40% in this trial.

There is growing consensus about the fact that objective antitumour response may not be the most important end point to be used to evaluate the efficacy of immunotherapy. A low growth of the tumour, resulting from a sustained immunological attack, may also benefit the patient. Thus, survival and performance status may be more appropriate parameters to compare treatment outcomes. A recent study (Rosenberg et al, 1994) reported an actuarial 36-month survival rate of 34% in metastatic RCC patients treated with high-dose bolus rIL-2. In the present study, the estimated actuarial 36-month survival rate of mRCC patients was 51%, and the ten patients still living at the last observation showed a very good PS (equal to 0). These results summarize the impact of immunotherapy on the natural history of mRCC, as about 10% of mRCC patients have an expected 3-year survival rate (Bottiger, 1970). However, survival data from non-randomized phase II studies have little value because of selection bias, and randomized studies are necessary to compare survival data from different immunotherapeutic schedules.

In our patients, toxicity of rIL-2 was reduced to a level that was quite manageable on an ambulatory oncology ward. Toxicity was not related to the number of cycles, therefore we could repeat the treatment many times (up to 13 cycles to date) in all mRCC patients.

In summary, this study shows that repetitive cycles with very low doses of rIL-2 and rIFNα-2 bring about an objective response rate of 20% and a 44-month survival rate of 44%. Treatment-related toxicity is limited to WHO grades of severity 1 and 2 only.

### ACKNOWLEDGEMENTS

We wish to thank Dr A Mutti for critical evaluation of the manuscript and Mr G Gatti for performing statistical procedures.

### REFERENCES

Atzpodien J, Korfer A, Franks CR, Poliwoda H and Kirchner H (1990) Home therapy with recombinant interleukin-2 and interferon-α2b in advanced human malignancies. *Lancet* **335**: 1509–1512

Atzpodien J, Poliwoda H and Kirchner H (1991) Alpha-interferon and interleukin-2 in renal cell carcinoma: studies in nonhospitalized patients. *Semin Oncol* **18** (suppl. 7): 108–112

Bottiger LE (1970) Prognosis in renal carcinoma. *Cancer* **26**: 780–787

Caligiuri MA (1993) Low-dose recombinant interleukin-2 therapy: rationale and potential clinical applications. *Semin Oncol* **20** (suppl. 9): 3–10

Caligiuri MA, Zmuidzinas A, Manley TJ, Levine H, Smith KA and Ritz J (1990) Functional consequences of interleukin 2 receptor expression on resting human lymphocytes. Identification of a novel natural killer cell subset with high affinity receptors. *J Exp Med* **171**: 1509–1526

Fossa SD, Kramar A and Droz JP (1994) Prognostic factors and survival in patients with metastatic renal cell carcinoma treated with chemotherapy or interferon-α. *Eur J Cancer* **30A**: 1310–1314

Lenardo MJ (1991) Interleukin-2 programs mouse αβ T lymphocytes for apoptosis. *Nature* **353**: 858–861

Lissoni P, Barni S, Ardizzola A, Andres M, Scardino E, Carbellini P, Della Bitta R and Tancini G (1993) A randomized study of low-dose interleukin-2 subcutaneous immunotherapy versus interleukin-2 plus interferon-alpha as first line therapy for metastatic renal cell carcinoma. *Tumori* **79**: 397–400

Miller AB, Hoogstraten B, Staquet M and Winkler A (1981) Reporting results of cancer treatment. *Cancer* **47**: 207–214

Palmer PA, Vinke J, Philip T, Negrier S, Atzpodien J, Kirchner H, Oskam R and Franks CR (1992) Prognostic factors for survival in patients with advanced renal cell carcinoma treated with recombinant interleukin-2. *Ann Oncol* **3**: 475–480

Rosenberg SA, Lotze MT, Muul LM, Chang AE, Avis FP, Leitman S, Linehan WM, Robertson CN, Lee RE, Rubin JT, Seipp CA, Simpson CG and White DE (1987) A progress report on the treatment of 157 patients with advanced cancer using lymphokine-activated killer cells and interleukin-2 or high dose interleukin-2 alone. *N Engl J Med* **316**: 889–897

Rosenberg SA, Yang JC, Topalian SL, Schwartzentruber DJ, Weber JS, Parkinson DR, Seipp CA, Einhorn JH and White DE (1994) Treatment of 283 consecutive patients with metastatic melanoma or renal cell cancer using high-dose bolus interleukin 2. *JAMA* **271**: 907–913

Schneekloth C, Korfer A, Hadam M, Hanninen EL, Menzel T, Schomburg A, Dallmann I, Kirchner H, Poliwoda H and Atzpodien J (1983) Low-dose interleukin-2 in combination with interferon-α effectively modulates biological response in vivo. *Acta Haematol* **89**: 13–21

Sondel PM, Kohler PC, Hank JA, Moore KH, Rosenthal NS, Sosman JA, Bechhofer R and Storer B (1988) Clinical and immunological effects of recombinant interleukin 2 given by repetitive weekly cycles to patients with cancer. *Cancer Res* **48**: 2561–2567

Vlasveld LT, Hekman A, Vyth-Dreese FA, Rankin EM, Scharenberg JGM, Voordouw AC, Sein JJ, Dellemijn TAM, Rodenhuis S and Melief CJM (1993) A phase I study of prolonged continuous infusion of low dose recombinant interleukin-2 in melanoma and renal cell cancer. Part II: immunological aspects. *Br J Cancer* **68**: 559–567

Vuoristo M, Jantunen I, Pyrhonen S, Muhonen T and Kellokumpu-Lehtinen P (1994) A combination of subcutaneous recombinant interleukin-2 and recombinant interferon-α in the treatment of advanced renal cell carcinoma or melanoma. *Eur J Cancer* **30A**: 530–532

West WH, Tauer KW, Yannelli JR, Marshall GD, Orr DW, Thurman GB and Oldham RK (1987) Constant infusion recombinant interleukin-2 in adoptive immunotherapy of advanced cancer. *N Engl J Med* **316**: 898–905

Wiebke EA, Rosenberg SA and Lotze MT (1988) Acute immunologic effects of interleukin-2 therapy in cancer patients: decreased delayed type hypersensitivity response and decreased proliferative response to soluble antigens. *J Clin Oncol* **6**: 1440–1449

© Cancer Research Campaign 1997